# Study on the Effect of 1-Butanol Soluble Lignin on Temperature-Sensitive Gel

**DOI:** 10.3390/polym10101109

**Published:** 2018-10-08

**Authors:** Pan Jiang, Yi Cheng, Sheng Yu, Jie Lu, Haisong Wang

**Affiliations:** School of Light Industry and Chemical Engineering, Dalian Polytechnic University, Dalian 116034, China; panjdemo@163.com (P.J.); chengyi@dicp.ac.cn (Y.C.); shengyu1342@163.com (S.Y.)

**Keywords:** 1-butanol, fractionation, industrial alkali lignin, temperature-sensitive gel

## Abstract

A protocol for the fractionation of lignin with 1-butanol as solvent has been proposed in order to improve the utilization of industry alkali lignin. 1-butanol soluble lignin (BSL) was used as a building block for temperature-sensitive hydrogel with *N*-isopropylacrylamide (NIPAAm) through graft polymerization. The result shows that 1-butanol fractionation is an effective method to improve the molecular weight homogeneity of lignin (PDI, 2.5 to 1.83) and increase the hydroxyl group content (0.585–1.793 mmol/g). The incorporation of BSL into the temperature-sensitive hydrogel can enhance the thermal stability and increase the hydrophobicity of the gel, which leads to a decrease in lower critical solution temperature (LCST). In addition, the compression strength, swelling ratio, and pore size of the gel can be adjusted by the dosage of lignin. This stimuli-responsive gel, with an LCST around 32 °C, is expected to be applied in the agricultural field as a pesticide carrier by stimulating release and absorption properties based on the change in natural environmental temperature.

## 1. Introduction

Lignin, the second most abundant natural terrestrial polymer on earth after cellulose, is considered the most promising raw material to replace petrochemical-based feedstock in light of its antioxidant and antimicrobial properties and its high carbon content [1,2,3]. To date, lignin is mainly obtained from pulp and paper companies and the bio-refinery industry as a byproduct at a rate of several million tons per year [4,5]. Despite its great abundance, most lignins are burned as a cheap source of energy to meet domestic energy consumption, and only a small amount of industrial lignins have been used to develop high-valued products, due to their intrinsic structural heterogeneity, poor solubility in common solvents, and wide distribution of molecular weights. Among them, the heterogeneity of lignins has become more severe in chemical pulping processes due to the incomplete impregnation of the chemicals in feedstock, which poses a challenge for applications where a constant molecular size is required [6,7,8]. To circumvent this problem, solvent fractionation was proposed by scientific researchers based on partial solubility of polymers into solvents. Firstly, the lignin is dissolved in a specific solvent, and a non-solvent, or a solvent with poor solubility of lignin is then added to precipitate the lignin. The obtained soluble lignin presents a narrower molecular weight distribution and higher reactivity. Along this line, several attempts have been made to further reduce the heterogeneity of lignin [9,10,11,12]. All results indicate that solvent fractionation is a promising method to obtain lignin with high purity and narrower molecular weight distribution. There is an increasing interest in utilizing lignin with high quality to develop novel lignin-based functional material for the purpose of exploiting the substantial potential value of lignin.

Stimuli-responsive hydrogel, also known as smart hydrogel, has attracted a great deal of interest. The stimuli-responsive hydrogel has three-dimensional networks composed of crosslinked polymer chains that undergo reversible phase transition in response to external stimuli (e.g., temperature [13,14,15,16], CO_2_ [17], and pH [18]). *N*-isopropylacrylamide (NIPAAm) is a vinyl monomer with a hydrophilic and hydrophobic balance, thus rendering sensitive behavior to temperature around 32 °C, which is close to human body temperature and is a natural environmental temperature. Therefore, NIPAAm is usually used as the start material for temperature-sensitive hydrogels and widely applied in drug release [19,20,21], sensors [22], cartilage repair [23], wastewater treatment [24], multiple response material [25,26], actuators [27], electronic devices [28], and glucose detection [29]. Here, we introduced lignin into the structure of poly(*N*-isopropylacrylamide) PNIPAAm and prepared a series of lignin-based stimuli-responsive gel. For the first step, high-quality lignin was prepared from industrial by-products by fractionation with 1-butanol. Subsequently, stimuli-responsive gel was synthesized through radical polymerization. The water absorption, thermal property, interior morphology, and the mechanical properties of the obtained gel were investigated in detail.

## 2. Materials and Methods 

### 2.1. Raw Materials and Chemicals

Industry alkali lignin (AL) was collected from Tranlin Forest & Paper Co. Ltd. (Liaocheng, Shandong, China). *N*,*N*′-Methylenebisacrylamide (MBA, AR, 98%) was purchased from the Tianjin Guangfu Fine Chemical Research Institute (Tianjin, China). Dimethyl sulfoxide (DMSO, AR), hydrogen peroxide (AR, 30%), and calcium chloride (Anhydrous, AR, 98%) were purchased from Kermel Chemical Reagent Co., Ltd. (Tianjin, China). *N*-Isopropylacrylamide (NIPAAm, 98%) and dialysis tube (*M*_w_ 500, USA) were purchased from Aladdin. Unless otherwise specified, all reagents were used as received.

### 2.2. Lignin Purification and Fractionation

Raw alkali lignin was firstly dissolved in deionized water (m/m, 1:10), filtered to remove the insoluble solid impurities. The pH of filtrate was adjusted to 2–3 by sulfuric acid solution (0.2 mol/L), and the filtrate was then kept in a water bath (70 °C) until lignin was aggregated into lumps. Secondly, the solid lignin was transferred into a dialysis tube and immersed in sufficient deionized water. The deionized water was refreshed every 12 h until the system became neutral. Finally, the solution was filtered and freeze-dried to obtain the purified alkali lignin (PL).

PL (1 g) was mixed with 1-butanol (5 mL) at ambient temperature, and the mixture was shaken in a constant temperature oscillator for 2 h at 90 rpm to promote its dissolution. After that, the solution and the insoluble PL were completely separated by centrifugation (3 × 6000 rpm). Finally, the supernatant solution was vacuum-dried at 75 °C to a constant weight to obtain 1-butanol soluble lignin (BSL).

### 2.3. Synthesis of Temperature-Sensitive Gel

Briefly, BSL and 1.2 g of NIPAAm with various mass ratios (g/g, 1:24, A; 1:12, B; 1:8, C) were completely dissolved in 2 mL of DMSO to form a homogeneous solution in the flask. Calcium chloride powder (0.2 g), hydrogen peroxide (5 wt % respect to BSL), and MBA (0.017 g) were added into the solution successively. After stirring homogeneously, the solution was bubbled with N_2_ to remove air. The solution was then sealed and placed into a water bath (70 °C) for 12 h under an N_2_ atmosphere for the gelation process. The formed wet gels were soaked in DMSO, and the liquid was refreshed every 12 h until it became transparent. DMSO was then replaced with deionized water to realize solvent exchange, and the wet gels were kept in deionized water for five days to remove any remaining DMSO. Finally, the cryogels were obtained after freeze drying.

### 2.4. Characterization of Lignin

Fourier-transform infrared spectroscopy (FTIR) spectra of samples were recorded using a Frontier Spectrometer (JASCO, Tokyo, Japan) in the range from 4000 to 400 cm^−1^ with a resolution of 4 cm^−1^ and 40 scans. Each sample was prepared with potassium bromide pellets. Glass transition temperature (*T_g_*) of lignin was determined using a differential scanning calorimeter (DSC250, TA, New Castle, DE, USA) linked to an aluminum pan, which runs at a rate of 10 °C/min from 0 to 250 °C, continually flushed with nitrogen at a flow of 40 mL/min, referenced against an empty pan. The intersection point of the tangent line at the inflection point on the heat flow curve was assigned as *T_g_.* A thermogravimetric analyzer (TGA, TA Q50, New Castle, DE, USA) was used to investigate the thermal stability of the samples. The samples were heated at a heating rate of 10 °C/min from 50 to 700 °C in a nitrogen atmosphere at a flow of 40 mL/min.

The molecular weight of lignin, including weight average molecular weights, number average molecular weight, and polydispersity, were determined by GPC instrument (Waters 1515/2414, Milford, MA, USA), equipped with a refractive index detector and two cascaded PL–gel columns at 35 °C with tetrahydrofuran (THF) as the mobile phase at a rate of 1.0 mL/min. In order to prevent the intermolecular association and improve the solubility of lignin, lignin samples were acetylated before molecular weight measurement. Lignin samples of 300 mg were subjected to acetylation after being dissolved in a 15 mL acetic anhydride/pyridine (2:1, *v*/*v*) mixture and kept in a cool and ventilated environment under an anaerobic atmosphere for 72 h. Acetylated lignin was precipitated and washed by diethyl ether (3 × 200 mL). Dried acetylated lignin was then dissolved in THF (10 mg/mL), filtered for analysis. Calibration was performed using polystyrenes with a molecular mass range from 580 Da to 3250 KDa.

NMR determination for lignin was performed in a Bruker AVIII 400MHz spectrometer (Bruker Daltonic Inc., Bremen, Germany). ^31^P NMR analysis drew on previous literature reports with minor modifications [30,31]. Briefly, 25 mg lignin samples were dissolved in 0.5 mL anhydrous pyridine/CDCl_3_ solvent (1.6:1, *v*/*v*) under stirring. A 0.1 mL internal standard solution including 10.25 mg/mL cyclohexanol in anhydrous pyridine/CDCl_3_ solvent (1.6:1, *v*/*v*) and a 0.1 mL relaxation reagent solution containing 5.1 mg/mL chromium (III) acetylacetonate in anhydrous pyridine/CDCl_3_ solvent (1.6:1, *v*/*v*) were added. Successively, 0.1 mL of 2-chloro-4,4,5,5-tetramethyl-1,3,2-dioxaphospholane (TMDP) were added into the mixture, which was then shaken for 2 h. Finally, phosphitylated lignin samples were transferred into NMR tube for analysis.

### 2.5. Characterization of Gel

The lower critical solution temperature (LCST) of the gels was determined using a differential scanning calorimeter (DSC250, TA, USA) linked to an aluminum pan, which runs in the temperature range from 0 to 40 °C for LCST, and continually flushed with nitrogen at a flow of 40 mL/min. Values were referenced against an empty pan. The intersection of the epitaxial line of the base line and the curved part of the heat flow change line was assigned as LCST. A thermogravimetric analyzer (TGA, TA Q50, New Castle, DE, USA) was used to investigate the thermal stability of the samples. The samples were heated at a heating rate of 10 °C/min from 50 to 700 °C in a nitrogen atmosphere at a flow of 40 mL/min.

The morphology was observed for samples sputter-coated with gold using a scanning electron microscope (SEM, JSM-7800F, Shimadzu, Tokyo, Japan) at an accelerating voltage of 20 kV. Elemental composition of samples was analyzed with the elemental analyzer (LeemanEA3000, Genoa, Italy). Specific surface area was measured with a Micromeritics ASAP 2020 surface areas and porosity analyzer (Micromeritics Instrument Corporation Ltd., Norcross, GA, USA). The mechanical properties of samples were tested using a universal material testing machine (INSTRON5960, Norwood, MA, USA) with a speed of 2 mm/min, the samples were compressed to 20% of the original height, and three specimens were measured for each sample.

Water absorption and desorption behavior of the gels were taken into consideration. Cryogels were soaked in deionized water at 20 °C. The weight of the wet gels was recorded every 2 h to obtain the absorption property. Gels were transferred to a water bath (40 °C) after the absorption equilibrium was achieved, and the gel weight was recorded every 2 h to study the desorption behavior. Equilibrium absorption ratio of gels were measured by immersing the cryogel in deionized water for 24 h within a temperature range from 40 to 20 °C, with intervals of 2 °C. All results were calculated with the formulas

Absorption ratio (%) = (*W*_a_ − W_c_)/*W*_c_ × 100 (1)

Desorption ratio (%) = (*W*_d_ − *W*_c_)/*W*_c_ × 100 (2)

Equilibrium absorption ratio (%) = (*W*_e_ − *W*_c_)/*W*_c_ × 100 (3)

where *W*_c_, *W*_a_, *W*_d_, and *W*_e_ represent the weight of the cryogel, the swelled gel, the deswelled gel, the equilibrium-swelled gel.

## 3. Results and Discussion

### 3.1. Lignin Fractionation and Characterization

The solubility of lignin in organic solvent depends on the Hildebrand solubility parameter (δ) of the solvent, which is the sum of three contributions: the dispersion component (∂_d_), the polar component (∂_p_), and the hydrogen bonding component (∂_h_) [6].

∂_t_^2^ = ∂_d_^2^ + ∂_p_^2^ + ∂_h_^2^. (4)

Lignin exhibits a maximum solubility in solvents with an δ-value close to their own [12]. Each single solvent has its own specific Hildebrand solubility parameter, which may not be close to that of lignin. Thus, the addition of hydroxylated solvents (such as water and low alcohols) can regulate the Hildebrand solubility parameter and improve the solubility of individual solvents [8,12,32].

In this work, 1-butanol (δ = 11.3, ∂_d_ = 16, ∂_p_ = 5.7, ∂_h_ = 15.8), containing an alkane chain that consists of four carbon atoms and a polar hydroxyl group, is used as a solvent. The dispersion force is the only force in the pure alkane solvents, while the polar hydroxyl could form hydrogen bond and polar interactions. Thus, the dispersion component, the hydrogen bonding component, and the polar component promote the dissolution of lignin in 1-butanol. This inspired us to study the lignin solubility from the point of view of the chemical structure and to predict the properties of fractionated lignin through solvent properties. Lignin monomer contains an alkane side chain with three carbon atoms and a hydroxyl group (the phenolic hydroxyl group and the aliphatic hydroxyl group) [2], which is similar to the chemical structure of 1-butanol. The similarity of the chemical structure between 1-butanol and lignin provides a possible dissolution. We infer that there will be differences in the chemical structure of certain functional groups between PL and BSL. Due to the incomplete dissolution of lignin in 1-butanol, we chose a single-step method to achieve solid–liquid separation.

### 3.2. Gel Formation and Characterization

**Figure 1:**
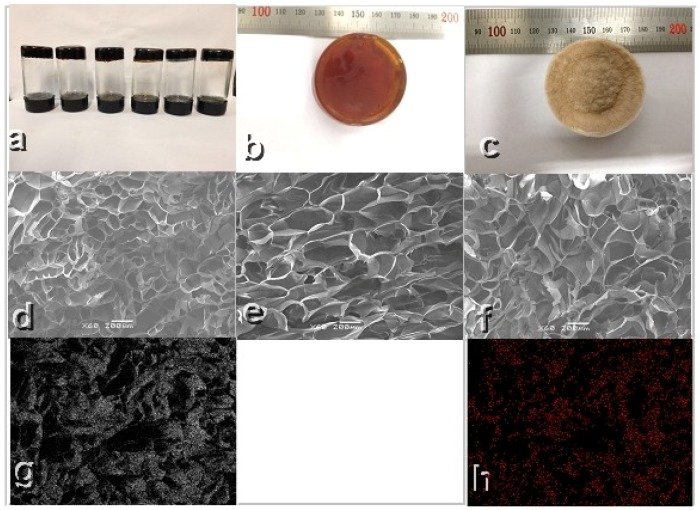
(**a**) Formed wet gel: 1:24 mass ratio; (**b**) swelling gel in deionized water: 1:24 mass ratio; (**c**) cryogel: 1:24 mass ratio. The SEM images of gels with (**d**) 1:24, (**e**) 1:12, and (**f**) 1:8 mass ratios. EDS images of 1:24 gel mass ratio: (**g**) carbon; (**h**) nitrogen.

Lignin contains several type of active functional groups—including hydroxyl (phenolic, aliphatic), carbonyl, carboxyl, carbon–carbon double bond, and methoxy groups—which enable chemical modification [33]. Especially, the phenolic hydroxyl can cause lignin to produce a star-like branched copolymer via graft reaction. In this work, the radical polymerization method was used to form a temperature-sensitive gel with fractionated lignin and NIPAAm. Firstly, a radical was created on the lignin structure mostly on the phenolic hydroxyl by a composite initiation system. The system was composed of hydrogen peroxide and anhydrous calcium chloride, which can initiate the polymerization of NIPAAm as well [34]. MBA was used as the cross-linking reagent to form a three-dimensional net structure. The formed wet gel (Figure 1a) was soaked in DMSO to remove excess reagents and impurities, and DMSO was then replaced by water to reach the swelling equilibrium state (Figure 1b). Finally, the cryogel was obtained after freeze drying (Figure 1c). In addition, several lignin-grafted polymers have been reported through atom transfer radical polymerization (ATRP) [35,36,37]. This method usually starts with esterification on lignin initiated by 2-bromoisobutyryl bromide to form an intermediate. The intermediate is used as a component of initiator mixture to complete the grafting reaction under the catalysis of 1,1,4,7,10,10-hexamethyl-triethylenetetramine and CuBr. The ATRP possesses multiple advantages, including higher grafting efficiency, experimental controllability, and lower coupling reactions between the lignin radicals [33].

### 3.3. Fourier Transform Infrared Spectroscopy (FTIR)

**Figure 2:**
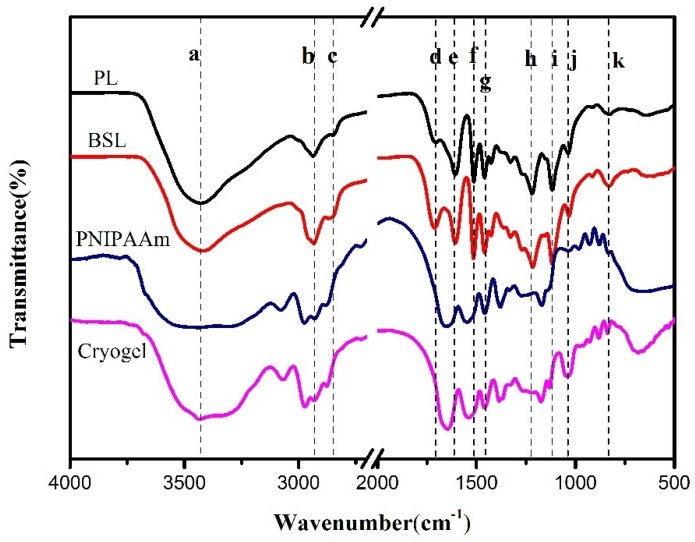
FTIR spectra of purified alkali lignin (PL), 1-butanol soluble lignin (BSL), poly(*N*-isopropylacrylamide) (PNIPAAm), and cryogel (mass ratio, 1:24).

To clarify the nature of the interactions between PL and solvent and gain further insights into the chemical composition of lignin fraction and gel, FTIR spectra were recorded (Figure 2). The recorded spectra of lignin samples were compared with the assignments found in other scientific reports [38,39,40]. As observed, both PL and BSL present a broad absorption centered at 3431 cm^−1^ (Peak a) with variable intensity that can be attributed to stretching vibrations of phenolic and aliphatic O–H groups. In addition, signals located at around 2933 cm^−1^ (Peak b) and 2843 cm^−1^ (Peak c) are assigned to stretching vibrations of C–H stretching vibration in methyl and methylene. Stretching vibration of C=O bonds of unconjugated carbonyl group are found around 1712 cm^−1^ (Peak d). The absorption band at 1610 cm^−1^ (Peak e), 1514 cm^−1^ (Peak f), and 1460 cm^−1^ (Peak g) identified in all lignin samples are assigned to aromatic skeletal vibration (1610 and 1514 cm^−1^) and aromatic methyl group vibrations (1460 cm^−1^). The peaks assigned to aromatic C–O stretching vibration are observed around 1218 cm^−1^ (Peak h). In addition, another characteristic signal of C–H bond in the aromatic rings (syringyl units) was presented at 1116 cm^−1^ (Peak i). Aromatic C–H in-plane deformation for guaiacyl type is centered at 1035 cm^−1^ (Peak j). Additionally, a band located at around 837 cm^−1^ (Peak k) is related to C–H, out of plane of the syringyl units. Evidently, the intensity of C–H stretching vibration of methyl or methylene and C=O bonds of unconjugated carbonyl group relative to the benzene ring in BSL are more visible than that in PL. The results confirm our assumptions that there are differences in the functional groups between PL and BSL.

The chemical structures of pure PNIPAAm and cryogel are provided. As shown in Figure 2, several characteristic peaks around 3400, 1653, and 1540 cm^−1^ were observed, which corresponds to N–H stretching vibration, C=O stretching vibration, and C–N–H deformation vibration (produced by N–H flexural vibration and C–N stretching vibration), respectively. C–H out of plane of the syringyl units in lignin and C–H stretching vibration of isopropyl on NIPAAm were observed at 837 cm^−1^ and 1374 cm^−1^, respectively. The results indicate that the graft reaction was successful.

### 3.4. ^31^P NMR

**Figure 3:**
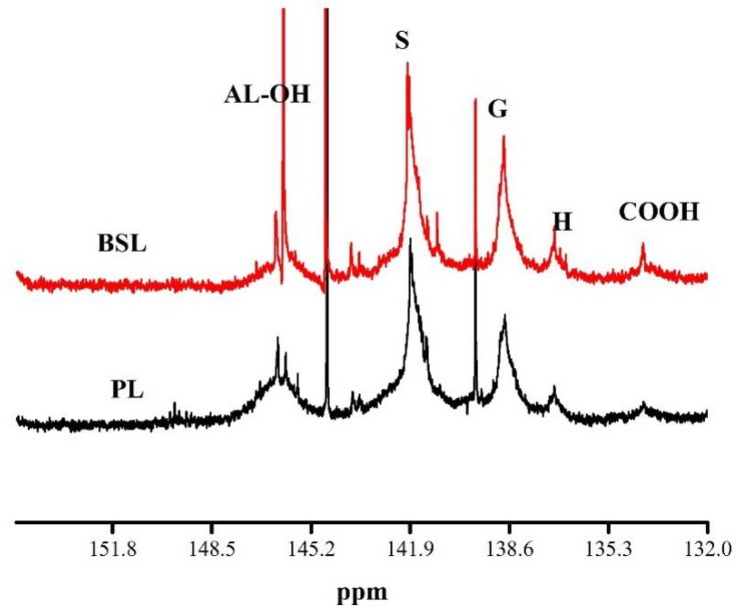
^31^P NMR spectra of PL, BSL.

The main chemical reaction activity of lignin comes from its hydroxyl group, including phenolic hydroxyl groups, aliphatic hydroxyl groups, and carboxyl hydroxyl groups. The aliphatic hydroxyl groups were originated from free hydroxyl groups in the side chain of lignin and residual carbohydrates [8]. In addition, the reactivity of phenolic hydroxyl groups coming from hydroxyl groups on the aromatic ring were influenced by high substitution on benzene rings [41]. The ^31^P NMR spectra of phosphorylated PL and BSL were recorded with cyclohexanol as an internal standard, and detailed quantitative analysis is presented in Table 1 and Figure 3. Based on the previous method [31], the peak of aliphatic OH (AL–OH) is located within the range of 145.4–150.0 ppm, and the phenolic hydroxyl groups include C5-substituted (S), guaiacyl (G) and P–hydroxyphenyl (H), ranging from 140.0–144.5, 139.0–140.2, and 137.0~137.8 ppm, respectively. In addition, the carboxyl hydroxyl groups (COOH) were calculated based on the range of 133.6–136.0 ppm.

As shown in Table 1 and Figure 3, all OH content was provided and was calculated relative to the internal standard (cyclohexanol). The content of aliphatic OH in BSL (0.590 mmol/g) was much higher than that in PL (0.075 mmol/g), which is illustrated by the interactions between lignin and solvent through dispersion force, polar force, and hydrogen bond. The amount of phenolic hydroxyl groups including C5-substituted, Guaiacyl, and P–hydroxyphenyl in BSL greatly increased after fractionation (C5-substituted increased from 0.236 to 0.731 mmol/g; Guaiacyl increased from 0.245 to 0.431 mmol/g; P–hydroxyphenyl increased from 0.029 to 0.041 mmol/g). In particular, it is worth noting that the phenolic hydroxyl groups content increased with the increase in the number of methoxy groups on the aromatic ring, which might be explained by the reason that wheat straw raw material is mostly comprised of s-type and g-type lignin. Moreover, C5-substituted phenolic OH content in BSL showed a higher ratio than that in PL due to the selective enrichment of solvent. The carboxylic acid OH in BSL was 0.016 mmol/g, which is much higher than that in PL. The result indicates that the 1-butanol treatment (or the extraction process) might induce ester hydrolysis, which leads to the generation of carboxylic acids or condensation, but the potential alteration of the overall physical characteristics (other than the decomposition behavior) is negligible [42].

### 3.5. Gel Permeation Chromatography (GPC)

To investigate the effect of fractionation on molecular weight and distribution of lignin, GPC analysis of the lignin samples was performed. The solubility of lignin might be affected by both molecular weight and chemical structure, so the fractionation products of different solvents will have different molecular weights and chemical structures [11]. As shown in Table 1, both number-average molecular weight (*M*_n_) and weight-average molecular weight (*M*_w_) decreased from 1953 and 4876 g/mol to 1825 and 3342 g/mol, respectively, after fractionation. Polydispersity indexes were also observed to decrease from 2.5 to 1.83. These results indicate that high-quality lignin with narrower molecular weight distribution and lower molecular weight can be obtained from solvent fractionation. Reports [42] have revealed that solvent treatment can generate oligomers with low molecular weight and generate chain fragments presenting in parent lignin. In addition, the lower molecular weight lignin with a shorter macromolecular chain makes it easier for the solvent to penetrate a highly branched 3D structure of lignin [6]. These results agree with the conclusion drawn from ^31^P NMR.

### 3.6. Glass Transition Temperature (T_g_)

**Figure 4:**
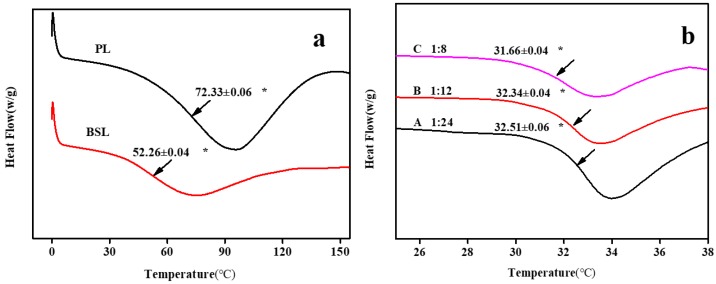
(**a**) Glass transition temperature (*T_g_*) of PL and BSL; (**b**) lower critical solution temperature (LCST) of gels; *n* = 3, mean values ± SD, * *p* < 0.01.

In an attempt to evaluate the effect of fractionation on glass transition *T_g_* of lignin, all samples were investigated via DSC analysis. To our knowledge, the glass transition temperature of lignin is not easy to be measured for its amorphous structure and another several factors including the presence of rigid phenyl groups in the main chain [43], crosslinking, inter-chain hydrogen bonding [44], molecular mass [45], thermal history, and solvent [46,47]. Figure 4a presents the *T_g_* of PL and BSL, obviously. BSL showed a lower *T_g_* (52.26 °C) than PL (72.33 °C), indicating solvent fractionation could bring a variation of *T_g_*. The decrease in *T_g_* could attribute to following reasons. Firstly, carbohydrate interacts with lignin through various chemical bond which limits molecular motion of lignin, the BSL with high purity contains lower carbohydrate allows more space for molecular movement thus leading to the decrease in *T_g_* [42]. In addition, aliphatic groups readily allow a free volume expansion and volatilization upon heating compared to the aromatic ring. The presence of higher aliphatic carbon atoms could decrease the *T_g_* of lignin by a free volume expansion.

### 3.7. Lower Critical Solution Temperature (LCST)

The lower critical solution temperature (LCST) of the gels is shown in Figure 4b. The phase transformation mainly depends on the interplay of PNIPAM intra-chain interaction and the interaction between water and PNIPAM. When the system temperature is lower than LCST, there is a strong hydrogen bond between the hydrophilic side chain and the water molecule. The polymer chain is hydrated and elongated, and the gel absorbs water and swells. Conversely, while the temperature of the system is higher than LCST, the hydrogen bond between the side chain and the water molecule decreases sharply, and the gel exhibits a dehydrated phase transition. The introduction of lignin enhanced the hydrophobicity of NIPAAm, which means that a lower temperature could break this balance. The LCST of the gels decreased with the increase in lignin content (A 1:24 to C 1:8; 32.51–31.66 °C), which is lower than that of pure NIPAAm (32.66 °C). Those results suggest that NIPAAm was successfully grafted onto lignin and the compound exhibited temperature-sensitivity at around 32 °C.

### 3.8. Elemental Composition of Lignin Fractions and Gel

To investigate the chemical composition of lignin and gel, elemental analysis was performed. As shown in Table 2, the nitrogen content in PL (0.625%) was higher than that in BSL (0.365%). To our knowledge, nitrogen comes from protein in the plant cell, which possesses low solubility in solvent. The result suggests that solvent fractionation can reduce nitrogen content in lignin and improve the purity of lignin. After graft reaction, NIPAAm leads to a marked increase in nitrogen to 9%. It can also be also seen from Table 2 that the fractionation lignin has higher carbon content (65.66%) than PL (62.725%), indicating a reduced carbohydrate residual content in the fractionation lignin. The carbohydrate residual possesses a lower level of carbon ratio compared with the lignin. The changes of carbon and nitrogen content have an effect on the fluctuation of the C/N ratio of the materials. The C/N ratio of BSL can reach up to 179.13, but in the gel, it is as low as 5.22. The improvement of lignin purity is also reflected in the decrease in sulfur content (0.555–0.379%). However, the graft reaction leads to an increase in sulfur in gel and the sulfur content increased with the increase in lignin ratio, which means a higher utilization of NIPAAm.

### 3.9. Thermogravimetric Analysis (TGA)

**Figure 5:**
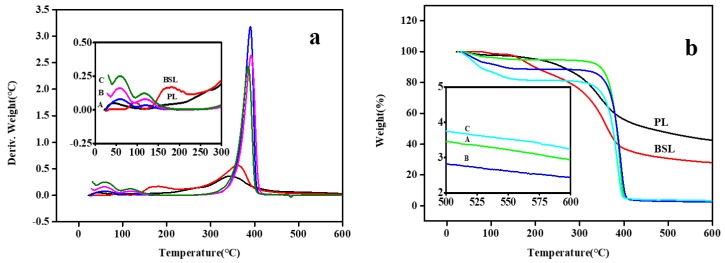
DTG (**a**) and TG (**b**) curves of PL, BSL, and the gel.

To investigate the effect of solvent fractionation on the thermal properties of lignin and the thermal stability of gel, TGA measurements were carried out. As shown in Figure 5, BSL showed a peak range from 150 to 250 °C and a higher onset temperature (360.57 °C) at which the sample weight loss became more apparent compared to PL (344.39 °C). This can be explained by decomposition of hydroxyl and the aliphatic functional group, which easily allows a free volume expansion and volatilization upon heating. Furthermore, the greater percentage of lignin content contains a lower amount of carbohydrates. BSL had a lower temperature (363.49 °C) at 50% weight loss and a lower char residue (27.94 wt %) at 600 °C compared with PL (458.86 °C; 42.46 wt %). This is due to the higher purity and various branches on the lignin structure, which leads to a wide range of degradation temperatures from 100 to 900 °C and a non-volatile above 800 °C. The decrease in char yield, from 42.46% of PL to 27.94% of BSL, is attributed to the thermal devolatilization of increased aliphatic groups.

Gels demonstrated a weight loss under 100 °C due to the evaporation of molecularly bound water. It is worth noting that the rate of mass change increased with the increase in lignin, which is attributed to the lower evaporation resistance of the increasing pore size (Figure 1D–F). The onset temperature of the gels exhibited no significant difference around 390 °C and showed a stable state over 400 °C. According to our previous work, highly condensed aromatic structures of lignin could help to improve the thermal stability of NIPAAm at 50% weight loss (130.7 °C). As shown in Table 3, the temperature of all gels at the weight loss of 50% was higher than 374 °C and the char residue remained above 2.44 wt %.

### 3.10. Interior Morphology and Mechanical Property

**Figure 6:**
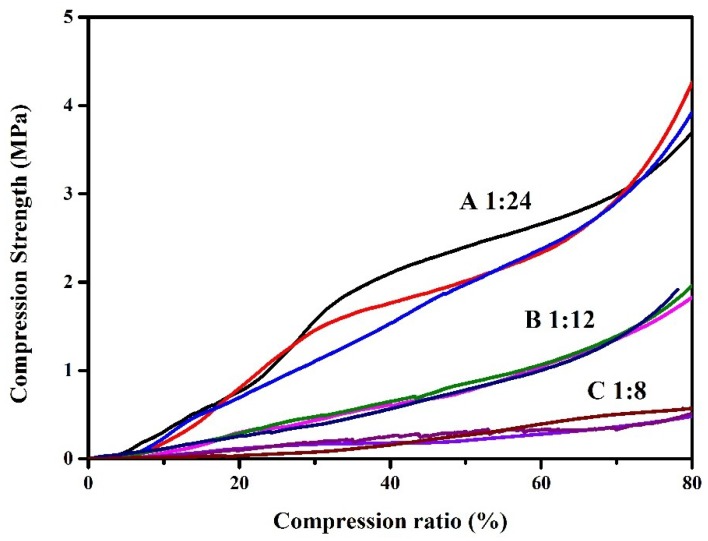
Compression strength of cryogels at 80% of the original height.

The interior morphology and mechanical properties of the cryogel are shown in Figure 1, Figure 6, and Table 3. The SEM image of gel with a low lignin dosage (Figure 1d) showed a dense pore distribution, and the pore size became increasingly large as lignin content increased (Table 3). However, gel with a much higher lignin content (Figure 1f) was brittle and showed an irregular distribution. The surface area of the gel was not more than 20 m^2^/g and presented a low mass ratio of lignin to NIPAAm of 1:12 (B). In this work, gels were prepared by freeze-drying, during which the wet gel was frozen and the solvent was then removed by sublimation at low pressures, leading to an increase in the amount of macropore and a decrease in surface area. The pore size of the gel increased as lignin content increased. When the pore size was much larger, the stress distribution was uneven, leading to cavity collapsing to form irregular internal topography and a complex structure, which causes an increase in the surface area (B–C, 13.96–19.11 m2/g) of the gels. In addition, the mechanical properties of the cryogels were characterized by a universal material testing machine. The increasing pore size leads to a decrease in the compression strength of the gel due to a decrease in the stress tolerance (3.96–0.52 MPa). Moreover, the EDS image of the gels (Figure 1g (carbon) and Figure 1h (nitrogen)) indicates that NIPAAm was dispersed evenly in the gel.

### 3.11. Water Absorption and Desorption Behavior

**Figure 7:**
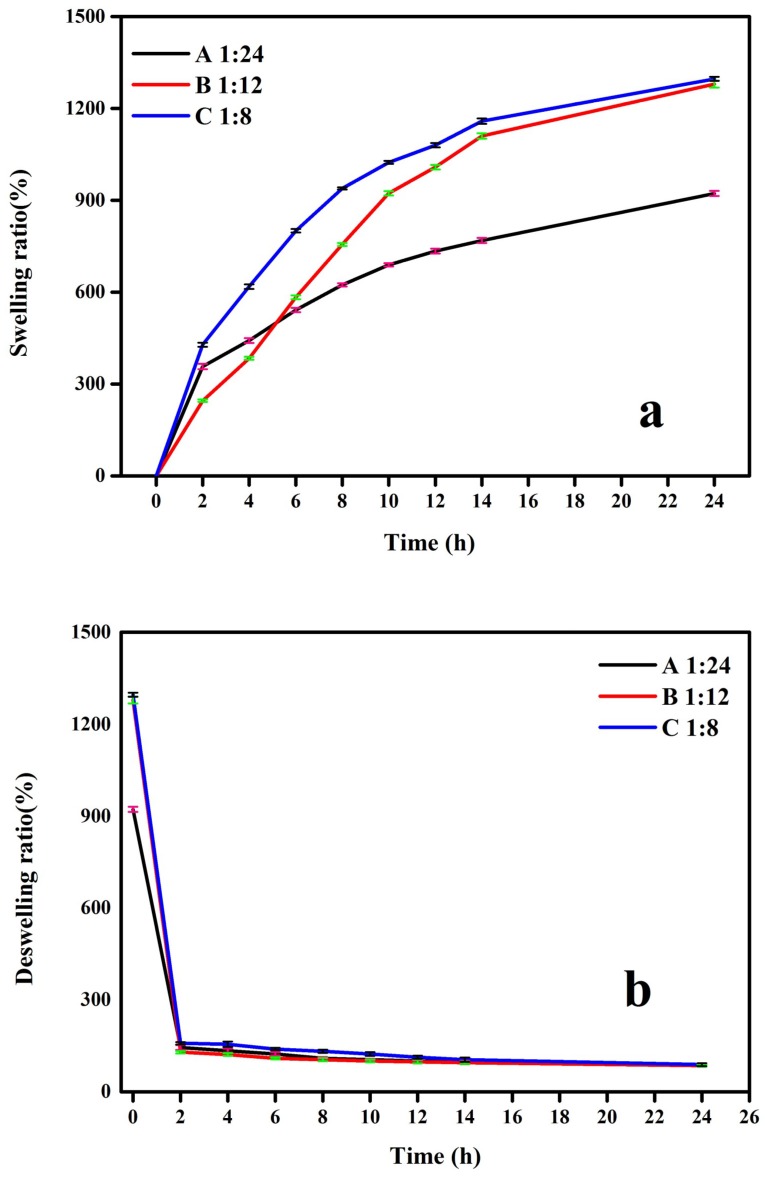

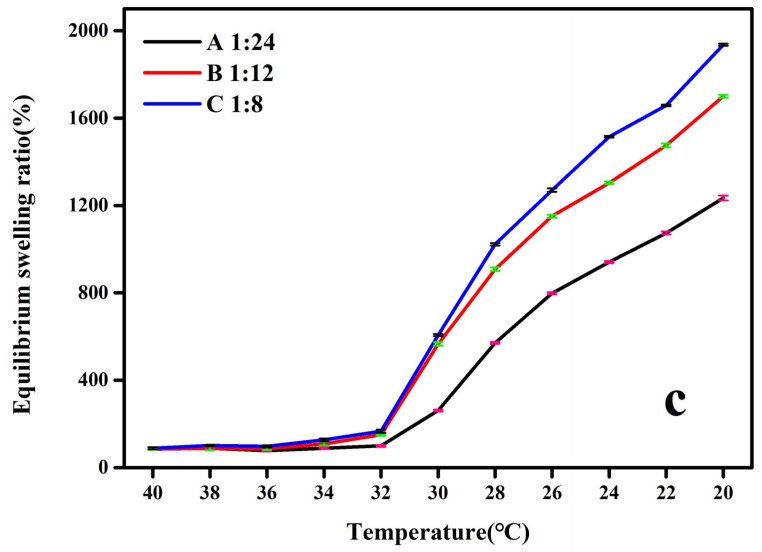
The swelling and deswelling behavior. (**a**) Swelling ratio at 20 °C; (**b**) deswelling ratio at 40 °C; (**c**) swelling ratio in the temperature range from 40 to 20 °C.

Cryogels were immersed in deionized water to study the influence of time on the absorption ratio. As shown in Figure 7a, gels were immersed in water at 20 °C to absorb water below the LCST (Figure 4). A (mass ratio of 1:24) and C (mass ratio of 1:8) exhibited a faster adsorption rate within 4 h compared to B (mass ratio of 1:12) due to their higher surface area. Over time, the adsorption ratio mainly depends on the pore size. A large pore size makes it easier for water molecules to enter the gel and thus increase the adsorption capacity. The maximum and minimum values at 24 h of adsorption capacity were 1298% (C, mass ratio of 1:8) and 930% (A, mass ratio of 1:24), respectively. The desorption ratio of the gels is shown in Figure 7b. All gels exhibited good temperature-sensitive properties. There was no significant difference in the residual water content when releasing water to a lower ratio state (A 85%, B 85%, C 88%) over 2 h above the LCST (at 40 °C). Figure 7c illustrates the equilibrium swelling ratio of the lignin-based gel at the temperature range from 40 to 20 °C with an interval of 2 °C for 24 h. It was found that the equilibrium swelling ratio had an obvious change when temperature dropped to 32 °C. The adsorption ratio of A, B, and C at 32 °C enhanced from 99%, 149%, and 166% to 261%, 566%, and 606% at 30 °C, which means that the LCST of gels is located within this range, and this corresponds well with the DSC result. In addition, for increased pore sizes, the maximum water absorption increases as lignin dosage increases.

## 4. Conclusions

1-butanol-dissolved lignin (BSL) was obtained from industry alkali lignin. 1-butanol treatment led to an increased concentration of methyl or methylene. A higher amount of the hydroxyl group and a lower amount of carbohydrates, sulfur, and nitrogen were also observed. It was evident that BSL exhibited a lower *T_g_* and thermal stability than that of raw lignin. The incorporation of BSL into the temperature-sensitive hydrogels can enhance the thermal stability and increase the hydrophobicity of the gels, which leads to a decrease in lower critical solution temperature (LCST). The freeze-dried gel showed a high compression strength and decreased with the increase in lignin dosage. On the contrary, a trend could be seen in the gel’s pore size: a higher lignin dosage corresponds to a larger pore size, while excessive lignin can cause aperture collapse. These stimulus-responsive gels, with an LCST of around 32 °C, close to a natural environmental temperature, are expected to be applied in the agricultural field as a pesticide carrier with stimulating release and absorption properties based on changes in natural environmental temperatures.

## Figures and Tables

**Table 1:** Quantification of the functional groups (mmol/g) in the lignins of PL and BSL using aquantitative ^31^P NMR method. Average relative molecular weight by GPC.

Samples	Aliphatic OH (mmol/g)	Phenols C5-Substituted (mmol/g)	Phenols Guaiacyl (mmol/g)	Phenols P–Hydroxyphenyl (mmol/g)	Carboxylic Acid OH (mmol/g)	*M*_w_	*M*_n_	PDI
	145.4–150.0 (ppm)	140.0–144.5 (ppm)	139.0–140.2 (ppm)	137.0~137.8 (ppm)	133.6–136.0 (ppm)			
PL	0.075	0.236	0.245	0.029	-	4876	1953	2.50
BSL	0.590	0.731	0.431	0.041	0.016	3342	1825	1.83

-: Not integrated.

**Table 2:** Elemental composition of lignin fraction and gel.

Samples	N (wt %)	C (wt %)	H (wt %)	S (wt %)	C/N	C/H
PL	0.625 ± 0.02	62.725 ± 0.20	5.781 ± 0.06	0.556 ± 0.02	100.005	10.851
BSL	0.365 ± 0.01	65.66 ± 0.33	6.397 ± 0.08	0.379 ± 0.01	179.130	10.265
A 1:24	11.235 ± 0.09	58.64 ± 0.25	9.647 ± 0.15	0.824 ± 0.03	5.220	6.079
B 1:12	9.705 ± 0.10	52.06 ± 0.24	8.802 ± 0.05	1.405 ± 0.02	5.365	5.913
C 1:8	9.775 ± 0.11	54.05 ± 0.28	9.103 ± 0.10	3.022 ± 0.05	5.530	5.938

**Table 3:** Thermal properties, compression strength, surface area, and pore size of samples.

Sample	Temperature at 50% Weight Loss (°C)	Onset Temperature (°C)	Char Residue at 600 °C (wt %)	Compression Strength (MPa)	Surface Area (m^2^/g)	Pore Size (nm)	Gel Yield (wt %)
PL	458.86	344.39	42.46	-	-	-	
BSL	363.49	360.57	27.94	-	-	-	
A	383.62	390.36	2.94	3.96 ± 0.23	17.18	2.35	93.5 ± 3.9
B	384.36	392.28	2.44	1.90 ± 0.06	13.96	2.44	86.6 ± 4.8
C	374.99	385.47	3.225	0.52 ± 0.03	19.11	2.46	80.5 ± 3.4
